# Genetically transforming human osteoblasts to sarcoma: development of an osteosarcoma model

**DOI:** 10.18632/genesandcancer.133

**Published:** 2017-01

**Authors:** Yi Yang, Rui Yang, Michael Roth, Sajida Piperdi, Wendong Zhang, Howard Dorfman, Pulivarthi Rao, Amy Park, Sandeep Tripathi, Carrie Freeman, Yunjia Zhang, Rebecca Sowers, Jeremy Rosenblum, David Geller, Bang Hoang, Jonathan Gill, Richard Gorlick

**Affiliations:** ^1^ Department of Pediatrics, Children’s Hospital at Montefiore, Albert Einstein College of Medicine, Bronx, NY, USA; ^2^ Department of Orthopaedic Surgery, Albert Einstein College of Medicine of Yeshiva University and Montefiore Medical Center, Bronx, NY, USA; ^3^ Department of Pathology, Albert Einstein College of Medicine of Yeshiva University and Montefiore Medical Center, Bronx, NY, USA; ^4^ Department of Pediatrics, Texas Children’s Cancer Center, Baylor College of Medicine, Houston, TX, USA; ^5^ Department of Molecular Pharmacology, Albert Einstein College of Medicine, Bronx, NY, USA; ^6^ Current affiliations: Department of Orthopaedic Surgery, Musculoskeletal Tumor Center, People’s Hospital, Peking University, Beijing, China; ^7^ Current affiliations: Pediatrics Administration, The University of Texas MD Anderson Cancer Center, Children’s Cancer Hospital, Houston, TX, USA

**Keywords:** osteosarcoma, mesenchymal stem cells, osteoblast

## Abstract

Osteosarcoma is the most common primary malignant bone tumor in children and young adults. Although histologically defined by the presence of malignant osteoid, the tumor possesses lineage multipotency suggesting it could be derived from a cell anywhere on the differentiation pathway between a mesenchymal stem cell (MSC) and a mature osteoblast. To determine if preosteoblasts (pOB) could be the cell of origin differentiated MSCs were transformed with defined genetic elements. MSCs and pOB differentiated from the same MSCs were serially transformed with the oncogenes hTERT, SV40 large T antigen and H-Ras. Assays were performed to determine their tumorigenic properties, differentiation capacity and histologic appearance. When subcutaneously implanted in immunocompromised mice, cell lines derived from transformed MSC and pOB formed tumors in 4 weeks. In contrast to the transformed MSC, the pOB tumors demonstrated a histological appearance characteristic of osteosarcoma. The cell lines derived from the transformed pOB only had osteogenic and chondrogenic differentiation potential, but not adipogenic ones. However, the transformed MSC cells and standard osteosarcoma cell lines maintained their tri-lineage differentiation capacity. The inability of the transformed pOB cell line to undergo adipogenic differentiation, may suggest that osteosarcoma is derived from a cell intermediate in differentiation between an MSC and a pOB, with partial commitment to the osteoblastic lineage.

## INTRODUCTION

Osteosarcoma is the most common primary malignant bone tumor, accounting for approximately 20% of all primary sarcomas in bone, and 2.4% of all malignancies in pediatric patients [1]. Pathologically osteosarcoma is defined by a malignant spindle cell which produces osteoid [2]. Although osteoid is pathognomonic of the diagnosis, tremendous variability exists in the predominant morphology which clinically is referred to as the histologic subtype. The conventional subtypes of osteosarcoma including the osteoblastic, chondroblastic and fibroblastic subtypes as well as the non-conventional morphologies including telangiectatic and small cell subtypes vary in their predominant histologic feature [3, 4]. The histologic subtype does not have a major influence on the clinical behavior or therapy effectiveness in this disease [4]. Molecular studies of osteosarcoma are greatly hampered by the enormous genetic instability that obscures the identification of genetic loci involved in osteosarcoma pathogenesis, and furthermore by the lack of benign precursors and no certainty as to the normal cellular counterpart or progenitor cells [5–7].

Although accumulated evidence has clarified the cancer cell of origin and mechanisms of transformation in epithelial and hematological malignancies, such aspects in many mesenchymal tumors are largely unknown. Theoretically, osteosarcoma could potentially be derived from a cell anywhere on the differentiation pathway between human mesenchymal stem cell (hMSC) and a mature osteoblast. It has been very difficult to define the cell of origin of osteosarcoma with the existing available data. The traditional view of osteosarcoma being derived from osteoblasts, mostly due to the presence of bony matrix osteoid, has been challenged by a number of recent studies suggesting that the cell of origin is a MSC [7–13].

In order to better understand the cell origin of osteosarcoma, our efforts have been directed towards developing a tumor which recapitulates osteosarcoma’s phenotype by introducing defined genetic elements, which had been described previously for transformation of other normal cell types [14–18]. Our initial study showed that two sarcomatous cell lines were established by introducing genetic alterations serially to transform hMSC into a malignant phenotype, but there was no osteoid production or osteoblast-like features observed in the neoplastic cells. These neoplastic cells could not be classified as an osteosarcoma [19]. Subsequently, with the hypothesis that introducing a genetic alteration inducing osteogenic differentiation may result in the desired phenotype, β-catenin, which may be involved in both tumor development and osteogenic differentiation, was introduced instead of H-Ras into partially transformed hMSC. The resulting cells did not produce tumors in mice and lacked the phenotype of fully malignant cells [20]. It remained unclear whether the failure to produce an osteosarcoma model was the result of MSC not being the cell of origin or once again incorrect selection of genes for cellular transformation.

In this study, a human osteosarcoma model was developed by introducing the same genetic alterations into pOB differentiated from hMSC. The neoplastic cells transformed from pOB, which only have bilineage (osteogenic and chondrogenic) differentiation potential, are phenotypically different from the derivatives of hMSCs. In xenograft experiments, the neoplasm showed a typical osteosarcoma histologic appearance and osteoid production could be easily identified.

## RESULTS

### hMSC cell culture

The hMSCs were grown on fibronectin-coated plates in MSC media and were allowed to mature to day 14 before they were passaged at a ratio of 1:3. The recovery rate and the uniformity of the cellular morphology increased with increasing passage number. In early passages, small groups of cells with a fibroblast-like morphology were observed which became more uniform in size and shape at passages 5 to 6 and higher (Figure S1A–C). All MSCs were karyotypically normal by spectral karyotyping when tested at passage 8 (Figure S1D). Flow cytometric analysis demonstrated that MSC cell surface markers were consistently and highly expressed. The MSC surface markers CD29, CD44, CD49e, CD73, CD90, CD105, and CD166 were expressed to levels greater than 95%, while the hematopoietic markers, CD34 and CD45, were less than 5% (data not shown). All hMSC cell lines showed the capacity to be committed, under proper conditions, towards adipogenic, chondrogenic and osteogenic phenotypes. (Figure S2A-D)

### Evidence for Osteoblast Differentiation prior to Transformation

Human MSCs induced with the osteogenic differentiation media on culture-day 14 were positive for ALP (Figure S2E) and Alizarin Red staining (Figure S2D), and the progression of osteogenic differentiation was confirmed by increasing levels of ALP activity of hMSCs in osteogenic media that reached the highest level on day 28 and was significantly higher than the level on day 0 (P < 0.01) (Figure S2F). Differentiation of hMSCs in osteogenic medium into pOB was further supported by mineralization of the extracellular matrix (ECM). The level of osteocalcin expression from hMSCs in osteogenic media on culture-day 28 was significantly higher than that of day 0 (P < 0.05) (Figure S3). Levels of calcium deposition per milligram protein in a group of hMSCs in osteogenic media on cell culture plates significantly increased from day 14 to day 28 (P < 0.01) (Figure S3).

### Establishment of Genetically Modified Pre-osteoblast and Mesenchymal Stem Cell Lines

hMSCs and pOB differentiated from the same hMSCs stably expressing hTERT, SV40 TAg and H-Ras were created serially through the use of independent selectable markers (neomycin, puromycin, and blasticidin, respectively) after transfection by viral constructs as described previously. For each transfection, parallel cultures were infected with empty vector specifying only a drug resistance gene as a control except the infection with H-Ras because of the CcdB suicide gene contained in the destination vector. The derivatives of hMSCs and pOB are listed in Table S2. The over expression of hTERT was confirmed by RT-PCR and functional telomerase activity was present *in vitro* assessed by TRAP assays. Expression of TAg and H-Ras were detected in MSC-TSR and OB-TSR through western blots (Figure S4). No distinguishable changes in cellular morphology, growth rate, and growth pattern among separate selected colonies were observed after viral transfection; therefore, the colonies were pooled together for further analysis.

### Changes in the Motility of the Transformed Cell Lines

There are many different kinds of cellular motility, including random migration, which can be measured by wound-healing assays (Figure S5) and haptotaxis, a cell movement towards an immobilized extracellular matrix (ECM) protein gradient, which is usually measured by a Boyden chamber system (data not shown). In our experiments, no significant changes were observed when comparing the random and haptotaxis migration capacity of MSC-TSR, pOB-TSR and mOB-TSR.

### Anchorage Independent Growth of the Transformed Cell Lines

**Figure 1:**
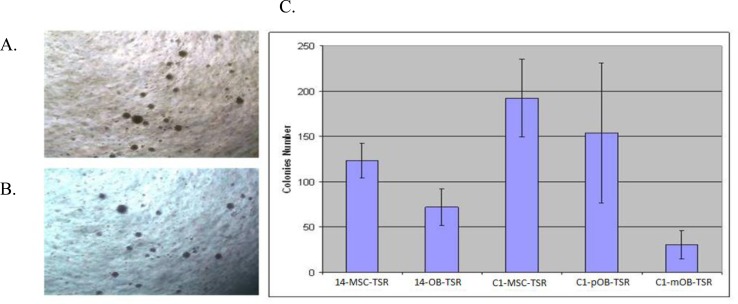
Anchorage independent growth in soft agar of transfected cell lines Anchorage independent growth was measured in (A) hMSC-TSR; (B) pOB-TSR; (C) Colony numbers of MSC-TSR and pOB-TSR were significantly greater than that of mOB-TSR (P < .01).

After transfection of H-Ras, cells lost contact inhibition when reaching 100% confluence, developing a multilayer growth pattern. Anchorage-independent growth of hMSC and its derivatives were measured through a colony forming assay in soft agar with the osteosarcoma standard cell line HOS used as a positive control. MSC-TSR and pOB-TSR formed variable numbers of colonies (Figure 1A-B). Colony numbers of MSC-TSR and pOB-TSR were significantly greater than that of mOB-TSR (P < .01) (Figure 1C).

### Tumorigenicity of the Transformed Cell Lines

**Figure 2:**
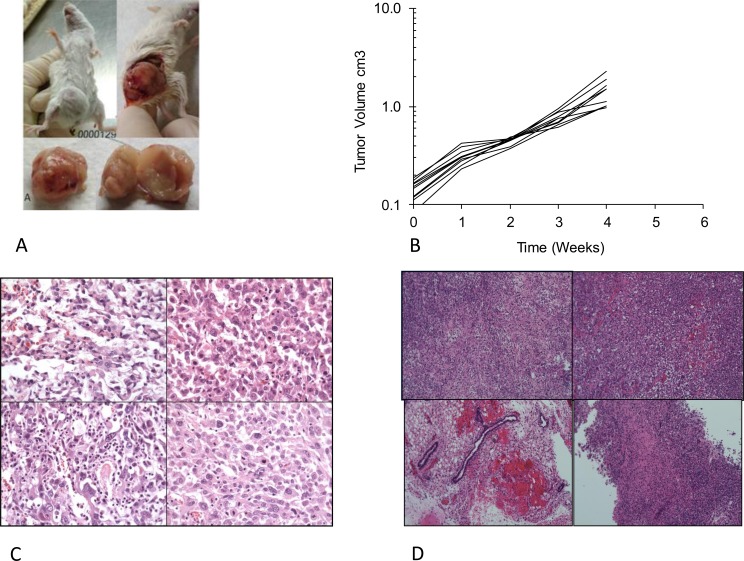
Tumorigenicity assays in SCID mice (A) Gross picture of hMSC-TSR and pOB-TSR cell lines implanted in mouse subcutaneously. (B) Tumors growth curve in mice after pOB-TSR cells injection. Once the size of the tumor reached to 1.7 cm in diameter or having 20% weight loss, the mouse was sacrificed according to the animal use protocol. (C-D) Histopathological findings of hMSC-TSR and pOB-TSR cell lines. Os, osteogenesis. T, tumor.

To examine the tumorigenic potential of the genetically modified hMSCs, pOB, and mOB *in vivo*, the transformed cells were injected subcutaneously into syngeneic 8-week-old female CB-17 SCID mice. All mice that received mOB-TSR survived during the observation period without apparent development of tumors. In contrast, all of the mice injected with MSC-TSR and pOB-TSR developed rapid growing tumors and died within 60 days (Figure 2A, B). Post-mortem examination revealed widely disseminated tumors accompanied by highly invasive lesions. In the MSC-TSR group, the tumors consisted of malignant spindle shaped cells without signs of osteoid formation (Figure 2C). In the pOB-TSR group, the tumors consisted of fascicles of spindle shaped cells with widespread distribution of a homogeneous eosinophilic (osteoid) material (Figure 2D). These histological features were diagnostic of osteosarcoma. All tumor cells were strongly positive for H-Ras immunoreactivity. The formation of H-Ras-positive cartilage in the tumors suggested that the injected cells contained chondrocytic progenitor cells.

### Differentiation Capacity of the Transformed Cell Lines

**Figure 3:**
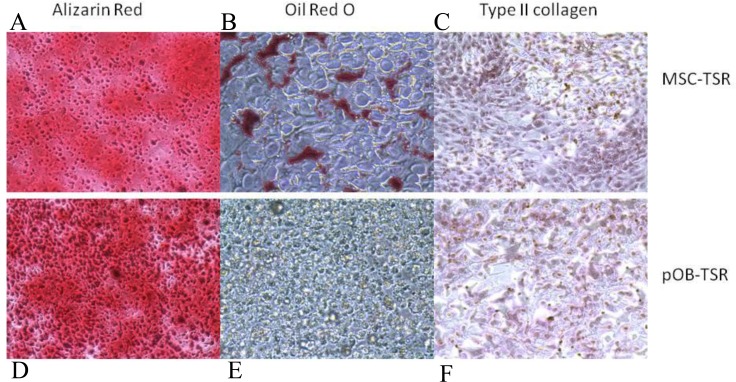
Changes in multilineage differentiation capacity in hMSC-TSR and pOB-TSR Osteogenic differentiation staining with Alizarin Red, adipogenic differentiation staining with Oil Red O, and chondrogenic differentiation immunohistochemical staining with type II collagen.

Human MSCs and pOB as described previously have and retain the ability to differentiate into three different mesenchymal lineages. Under optimal differentiation conditions, all genetically modified hMSCs were still able to undergo osteogenic, adipogenic and chondrogenic differentiation (Figure 3A, B, C), while the genetically modified derivatives of pOB only maintained bilineage (osteogenic and chondrogenic) differentiation potential (Figure 3D, E, F). To confirm the difference in the differentiation pattern of MSC-TSR and pOB-TSR cells, as well as to begin to elucidate the molecular basis of the difference in tumorigenesis between the two cell types, the expression of genes associated with differentiation were assessed in the MSC-TSR and the pOB-TSR cells. Furthermore, we tested a panel of 5 osteosarcoma standard cell lines as well as 5 patient derived cell lines. Among them, all the standard cell lines and four of patient derived cell lines maintained adipocyte differentiation capacity. The expression levels for adipogenic genes, including Pparg, Lpl and Fabp4, became elevated in MSC-TSR cells under adipogenic culture conditions. In contrast, the expression of these three genes was never detected in pOB-TSR cells, even under adipogenic culture conditions (data not shown). In contrast, among several osteogenic differentiation-related genes, the expression of Sp7, Runx2, Bglap, Ibsp and Alp were higher in pOB-TSR cells than in MSC-TSR cells (data not shown). These expression results support the view that the pOB-TSR cells are somewhat committed to osteogenic or chondrogenic and not adipogenic differentiation.

### Gene expression profiling

**Figure 4:**
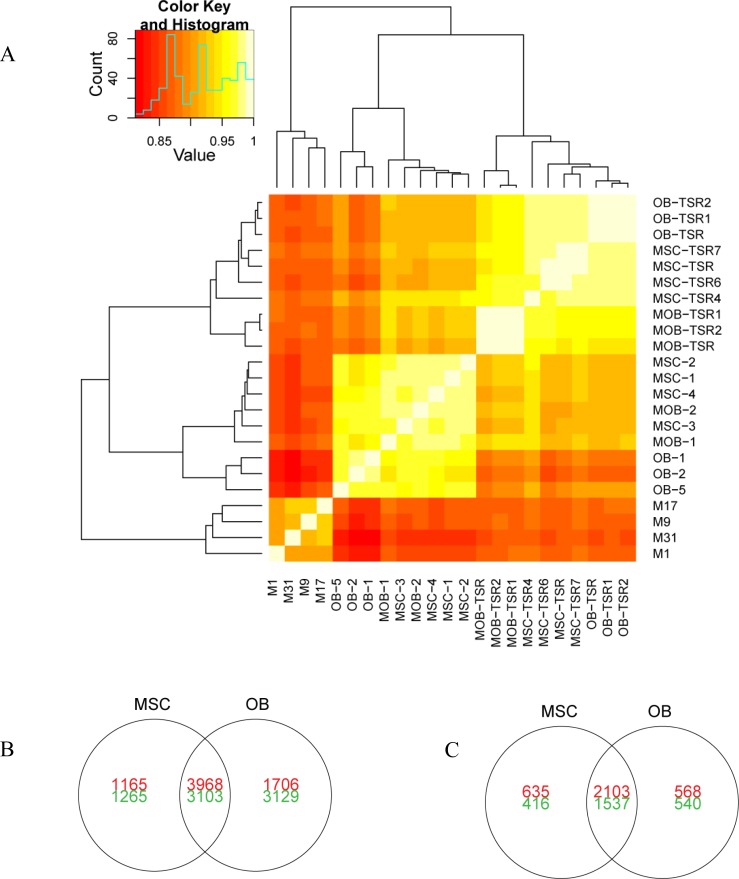
Gene expression differences between transformed cell lines, untransformed cell lines, and osteosarcoma xenografts (A) Unsupervised hierarchical clustering and heat map of gene expression correlation for untransformed cell lines, transformed cell lines, and osteosarcoma xenografts (M1, M9, M17, M31). Venn diagrams representing the number of genes differentially expressed (q-value <0.01) by transformed MSCs and pOBs as compared to (B) untransformed cell lines and (C) osteosarcoma xenografts. Red indicates increased expression and green indicates decreased expression.

To determine the gene expression differences underlying the differences in tumor histology and differentiation capacity of the transformed cell lines, the gene expression profiles of both transformed and untransformed cell lines were analyzed along with xenograft tumors. Transformed MSCs and pOBs have the most similar expression profiles based on unsupervised hierarchical clustering, which are distinct from osteosarcoma xenograft expression profiles (Figure 4A). Cell line transformation resulted in over 7,000 significantly differentially expressed genes in both cell lines (Figure 4B). A panel of seven genes among was selected and validated using real-time RT-PCR. Excellence correlation coefficient was found in two out of three specimens tested and moderate correlation was found in one sample (Table S1). Ingenuity Pathway Analysis of these genes indicated that many were involved with DNA damage repair and cell cycle regulation (Table S3). When comparing the gene expression profiles of the transformed cell lines and osteosarcoma xenografts (Figure 4C), genes from several cell signaling pathways are differentially expressed, including p53 signaling and the Wnt/β-catenin pathway (Table S3).

## DISCUSSION

Despite decades of clinical trials with multi-agent chemotherapy, and improved surgical approaches, survival outcomes for many patients with osteosarcoma still remain poor. Osteosarcoma is a heterogeneous malignancy, in which neighboring cells have varied and numerous chromosomal abnormalities and molecular aberrations [24–27]. This heterogeneity has made it difficult to identify optimal therapies based on underlying tumor biology. A laboratory model is needed to understand the events leading to the transformation of human cells to osteosarcoma, and the molecular aberrations driving the proliferation of the malignant cells. While multiple investigators have demonstrated the transformation of murine-derived cells to osteosarcoma, the current study demonstrates the development of tumors, with an osteosarcoma-like phenotype, from human-derived cells [9,28,29].

Over the past decade, researchers have utilized mesenchymal stem cells to develop *in vivo* models of sarcomagenesis [30, 31]. Mouse MSCs (mMSCs), when placed in long-term *in vitro* culture, select for clones with loss of cell cycle regulation [12, 32]. When these mMSCs are subcutaneously injected into immunodeficient mice they lead to the formation of sarcomas, including osteosarcoma [12, 13, 32, 33]. Human MSCs, unlike mMSCs, do not transform when cultured *in vitro* for extensive periods of time, possibly secondary to increased genetic stability [34]. Multiple studies have demonstrated the development of osteosarcoma from the transformation of various murine derived cells including MSCs, mesenchymal cells of limb buds, and pre-osteoblasts with the inactivation of INK4A/ARF or p53+/−Rb [7, 35–38]. Recent studies have also suggested an SV40 immortalized murine osteocyte cell line has the potential to form osteosarcoma-like tumors when injected into mice [39]. Manipulation of hMSCs with the introduction of various oncogenes, however, consistently yields undifferentiated sarcomas when subcutaneously injected in immunodeficient mice [19, 40, 41]. Rubio, et al, recently demonstrated that the bone microenvironment may play an essential role in the development of tumors with an osteosarcoma-like phenotype from p53^−/−^Rb^−/−^ hMSCs [42]. The model described in the current study generated osteosarcoma-like tumors from human pre-osteoblasts via the introduction of hTERT, SV40 large T antigen, and H-Ras, the same oncogenes that led to the development of undifferentiated spindle cell sarcomas when introduced in hMSCs. This suggests the differentiation stage specificity required to transform human cells to osteosarcoma and provides a possible explanation for the inability of prior studies to transform human cells to osteosarcoma.

**Figure 5:**
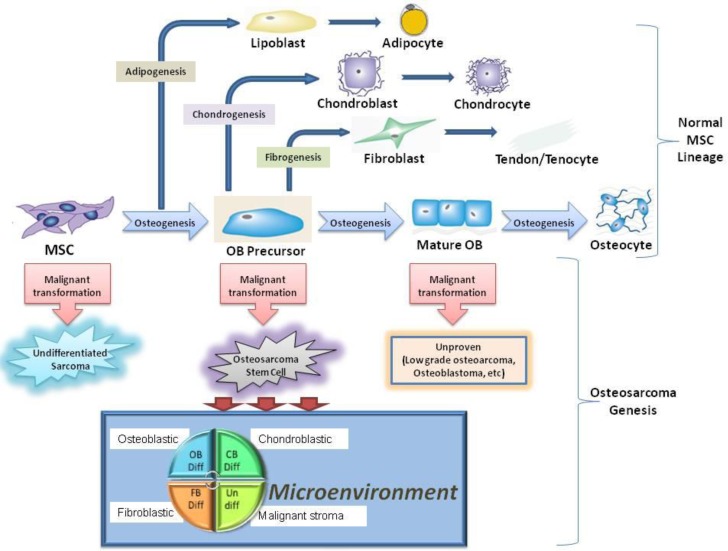
Schematic representation for the use of tumorigenic MSCs and pOBs as a model to study osteosarcoma genesis

The identification of the cancer-initiating cell has been a goal of investigators across malignancies. There has been significant debate over what is the cell of origin in osteosarcoma with many arguing the MSC is the cell of origin [7, 12, 42]. Others argue the cell of origin in osteosarcoma is already differentiated towards the osteoblast lineage, as suggested by studies demonstrating conditional knockout of p53/Rb in the pre-osteoblast lineage leads to the formation of osteosarcoma-like tumors [8, 29]. As described previously, the prior study demonstrated hMSCs transformed with hTERT, SV40 large T antigen and activated H-Ras subcutaneously injected into SCID mice do not form osteosarcoma, but rather formed a malignant spindle cell tumor that does not produce osteoid. While the current study demonstrates that human pre-osteoblasts can be transformed into osteosarcoma-like tumors, the pre-osteoblast is not likely the cell of origin in osteosarcoma. The majority of human osteosarcoma specimens have the ability to undergo tri-lineage differentiation specifically osteogenic, chondrogenic, and adipogenic differentiation [4]. In the current study, the transformed pOB-TSR cells retained osteogenic and chondrogenic differentiation capacity, however, the transformed cells no longer had the capacity to undergo adipogenic differentiation. Mohensy et al. demonstrated 89% of osteosarcoma cell lines have the capacity to undergo adipogenic differentiation [43]. While the stages of osteoblast differentiation have not been clearly delineated, it is known that early progenitor cells have tri-lineage differentiation capacity. As the progenitor cells mature, experiments suggest the cells first lose adipogenic differentiation capacity, and subsequently lose chondrogenic differentiation capacity (Figure 5) [19, 20, 30]. This theory is supported by studies demonstrating hMSCs transformed with hTERT, SV40, and β-catenin lose the capacity to be induced towards adipogenic differentiation; while the capacity to induce osteogenic differentiation in the transformed cells remain, and the capacity to induce chondrogenic differentiation is delayed [30]. This suggests the pre-osteoblast used in the current study is likely more mature than the cell of origin in osteosarcoma, as the transformed pre-osteoblasts have lost the capacity to undergo adipogenic differentiation. In addition, when the same genetic alterations were introduced into mature osteoblasts and injected into mice, no tumors formed, suggesting there is a stage in osteoblast differentiation in which more mature cells no longer have ability to form osteosarcoma. The current study findings demonstrating that transformed hMSCs produce malignant spindle cell tumors unable to produce osteoid and transformed pre-osteoblasts produce osteosarcoma unable to undergo adipogenic differentiation suggest the cell of origin in osteosarcoma exists in an intermediate differentiation stage between the MSC and pre-osteoblast [19]. This concept is supported by Rubio et. al’s data demonstrating p53−/− and Rb−/− MSCs derived from mouse bone marrow, when subcutaneously injected into SCID mice, form malignant spindle cell tumors consistent with leiomyosarcomas, however, when the MSCs are first placed in osteogenic differentiation media and then injected into immunodeficient mice they form osteosarcoma [28]. Studies assessing the transformation of pre-osteoblast cells to osteosarcoma currently define the stage of osteogenic differentiation based on the number of days MSCs are incubated in differentiation media, and not on cell phenotype [28]. This does not allow for the clear identification of the cell of origin, as cells do not uniformly differentiate on a defined schedule. Additional studies, inserting alternative combinations of oncogenes into hMSCs differentiated towards the osteoblast lineage, should be conducted to confirm the importance of differentiation stage towards the development of osteosarcoma.

The current study provides a laboratory osteosarcoma model utilizing transformed human cells and provides novel insight into the cell of origin in osteosarcoma. In summary the study suggests that the MSC is not the likely cell of origin in osteosarcoma and some degree of osteoblastic differentiation is required to transform human cells to develop osteosarcoma *in vivo*. The study also demonstrates that the cell of origin in osteosarcoma is likely more immature than the pre-osteoblast stage. Additional studies are needed to better define the stages of osteoblast differentiation in order to help identify the cell of origin in osteosarcoma.

## MATERIALS AND METHODS

### Cell Culture and Characterization of phenotypes

Three normal healthy human bone-marrow-derived mesenchymal stem cells were used in this study. BM-MSC14 (M14) was obtained from a patient with acute lymphocytic leukemia in remission treated at the Children’s Hospital at Montefiore, Bronx, New York, with the parents/patients written informed consent and in accordance with a research protocol approved by the Montefiore Medical Center Institutional Review Board. MSC-L1 (ML1) and MSC-L2 (ML2) were purchased from Lonza (Allendale, NJ). All cells were characterized either at passage 5 or 7 through Fluorescence Activated Cell Sorting (FACS) analysis. The phenotypes were uniform among all the different cells tested and in agreement with those reported for MSCs, that is, CD90, CD105, CD166, HLA-A/B/C positive (>95%), and CD34, CD 45, CD31, CD80 and HLA-DR negative (<5%). In addition, spectral karyotyping for hMSC cell lines were performed eight passages after their derivation as described below. Furthermore, all hMSC cell lines were tested for their multipotency under proper conditions towards adipocytes, chondrocytes and osteocytes. The hMSCs and their transforming derivatives were cultured in mesenchymal stem cell growth media (Lonza), as previously described [19]. All hMSCs were induced to osteogenic differentiation for 4-weeks. Differentiated pOB were cultured in osteoblast growth media (Lonza) at 37°C with 5% CO2. A standard mature osteoblast cell line was also purchased from Lonza and cultured under the same condition as pOB. Data for all of the cell lines are listed in Table1.

Alizarin Red staining was performed every 7 days using a standard protocol. Briefly, cells were fixed in ice cold 70% alcohol for 15 minutes and stained for 10 minutes with 2% Alizarin red (Sigma, St. Louis, MO). Alkaline Phosphatase (ALP) activity, osteocalcin expression and calcium content were monitored on culture days 14 and 28. At each experimental end point, cells in six-well plates were washed twice in DPBS, lysed with RIPA buffer on ice for 0.5 hours. Protein solutions were centrifuged at 2,000g for 15 minutes at 4°C. The supernatants were stored at −20°C for the analysis of protein content and cell pellets were stored for calcium content analysis. The quantification of protein in cell lysates was performed according to the manufacturer’s instruction (Bio-Rad Protein assay kit; BIO-RAD, Hercules, CA). The absorbencies were read at 595 nm in duplicate using a microplate spectrophotometer (BIO-RAD, Hercules, CA). Total protein was quantitated on the same samples as the ALP activity and osteocalcin measurements. The cell protein solutions were assayed for ALP activity using a TRACP and ALP Assay Kit (Takara Bio Inc., Shiga, Japan) according to the manufacturer’s instructions. Quantification of osteocalcin was performed according to the manufacturer’s instructions using the Gla-type Osteocalcin ELISA kit (Takara Bio Inc.). For the analysis of calcium deposition, the cell pellets were washed twice in DPBS. Excess fluid was removed using absorbent filter paper and specimens were minced into small pieces with two scalpels and then incubated in 0.5 M HCl at 37°C for 12 hours. The solutions were then centrifuged at 13,000g for 5 minutes and the supernatants were collected and maintained at −20°C for the analysis. Calcium content in the cell lysate was quantified spectrophotometrically with StanBio Calcium Liquicolour Kit (Stanbio, Boerne, TX) according to the manufacturer’s instructions. The absorbance of the samples was read at A575 nm using a microplate spectrophotometer (BIO-RAD). The calcium concentration was calculated from a standard curve generated from a serial dilution of a calcium standard solution. ALP activity, osteocalcin expression and calcium content were reported as each value per milligram total cellular protein (/mg protein).

### Plasmid and retroviral gene transfer

Plasmid construction and virus-induced transfections were carried out as previously described [19]. Briefly, hTERT, SV40 and H-Ras were serially transfected into hMSCs, mature osteoblast and pOB differentiated from the hMSCs. Following 24 hours of co-culture, drug selections of infected hMSCs and osteoblasts were performed with 100 μg/mL G418, 0.5μg/mL puromycin, and 2 μg/mL blasticidin, respectively. Expression of hTERT was measured by the RT-PCR, telomeric repeat amplification protocol (TRAP) and TeloTAGGG Telomere Length Assay. Expression of SV40 TAg and H-Ras were measured by Western blot.

### Spectral Karyotyping (SKY)

The cocktail of human chromosome paints was obtained from Applied Spectral Imaging (ASI, Vista, CA). Hybridization and detection were carried out according to the manufacturer’s protocol, with slight modifications [21]. Chromosomes were counterstained with DAPI. For each case, a minimum of 10 metaphase cells was analyzed by SKY. Images were acquired with a SD300H Spectra cube (ASI) mounted on a Zeiss Axioplan II microscope using a custom designed optical filter (SKY-1) (Chroma Technology, Brattleboro, VT), and analyzed using SKY View 2.1.1 software (ASI, Vista, CA). Breakpoints on the SKY-painted chromosomes were determined by comparison of corresponding DAPI banding of the same chromosome. By this method, we were able to define the breakpoints on *add* and *der* chromosomes but were unable to assign the precise breakpoints of chromosomal segments from partner chromosomes that generated the *add* or *der* chromosomes. A breakpoint was considered recurring if identified in 2 or more cases. Clonal chromosomal abnormalities identified by SKY/G-banding were described according to ISCN (2009).

### Cell Proliferation and Motility and Migration Assays

Cell proliferation was measured using the MTT reagent (Roche Diagnostics, Mannheim, Germany) per manufacturer’s protocol. Motility (random migration) was measured by a wound healing assay as described previously [19]. Cells were cultured in serum-free medium 24 hours before creating wounds. Photos were taken every 8 hours until 48 hours. Migration (haptotaxis) was measured using the QCM Quantitative Cell Migration Assay (Chemicon, Temecula, CA). Cells were serum starved 24 hours before seeding into Boyden Chambers. Cells that migrated to the outside of the chamber were stained and extracted. Absorbance at 570 nm was measured using a microplate spectrophotometer (BIO-RAD). All assays were performed in triplicate.

### Soft Agar Assays

An anchorage-independent growth assay was performed using soft agar as a culture media. A base layer of 0.5% DMEM agar was placed onto 35-mm plates. Cells were seeded at a density of 5 × 10^3^ cells/plate in 0.35% top agar containing DMEM and 10% FBS. Two mL of DMEM medium was added the next day when the agar was solid. Media was changed every 3 days to 4 days. After 3 weeks, the plates were stained with 0.5 mL of 0.005% crystal violet and colonies were counted. All cell lines were assayed in triplicate.

### Subcutaneous and Orthotopic Tumorigenicity Assays

All animal care and procedures were performed in accordance with a protocol approved by the Institutional Animal Care and Utilization Committee of the Albert Einstein College of Medicine of Yeshiva University. For xenograft experiments, 2 × 10^5^ cells in suspension were mixed with matrigel and then injected orthotopically or subcutaneously into syngeneic 8-week-old female CB-17 SCID mice (Taconic, Germantown, NY). Tumors that formed were removed and subjected to routine histologic staining and interpretation by a pathologist (H.D.). A total of ten mice were used for each cell line injection. Mice were monitored every day after injection and the size of tumor formed were measured every week over the observation period (60 days). Animals were humanely euthanized by CO_2_ asphyxiation when they met the following clinical criteria, weight loss of 20% or moribund activity or tumor diameter in any direction of 1.7cm. Since there was no drug treatment involved, neither analgesics nor anesthetics were given to mice to minimize pain. Only alcohol swab was used to wipe down the injection site.

### Differentiation Assays

For induction of adipogenesis, chondrogenesis or osteogenesis, all hMSCs, pOB and their derivatives were incubated in Lonza MSC Differentiation Medium supplemented with appropriate materials, as described previously [19]. Differentiated cells were stained with Alizarin Red, Oil Red O and immunohistochemically with antibody to collagen type II (Santa Cruz, Santa Cruz, CA), which can stain calcium, fat, and type II collagen, respectively, to verify formation of osteoblasts, adipoblasts, and chondroblasts.

### Microarray Assays

Total RNA from osteosarcoma xenografts, transformed and untransformed cell lines was extracted with RNeasy Mini Spin columns (Qiagen, Valencia, CA), and analyzed using Affymetrix Gene 1.0 ST Chip (Affymetrix, Santa Clara, CA). Arrays were normalized and batch corrected using the RMA module in package ‘affy’ and the ComBat module available in package ‘sva’, respectively [22, 23]. Expression differences between the various groups were identified using the ‘limma’ package (Bioconductor) [23]. All analyses were performed using R statistical software. Differentially expressed genes with a q-value <0.01 were retained for analysis with Ingenuity Pathway Analysis.

### Real Time Expression Analysis

Total RNA was extracted with RNeasy Mini Spin columns (Qiagen, Valencia, CA) and was subjected to reverse transcription using the Prime Script RT–PCR kit (Takara). Real-time RT–PCR analysis was performed using SYBR Premix Ex TaqII and Thermal Cycler Dice (Takara). Data are represented as the means ± s.d. from a minimum of three independent experiments. A panel of 7 genes involved in osteogenic, or chondrogenic, or adipogenic differentiation were selected. The sequences of the primers and probes have been described previously.

### Statistical Analysis

Differences in proliferation rate, colony formation in soft agar, and migration were compared between hMSC and its derivatives using chi-square and student t tests. Two-sided p values and 95% confidence intervals were calculated using a statistical software package (SPSS 20.0, Chicago, IL). A p-value of less than 0.05 was considered statistically significant.

## SUPPLEMENTARY FIGURES AND TABLES


