# Supplementary material for: Genetically transforming human osteoblasts to sarcoma: development of an osteosarcoma model
Source: Genes Cancer. 2017 Jan;8(1-2):484–94. doi: 10.18632/genesandcancer.133 (PMC5396624; doi:10.18632/genesandcancer.133)
Supplement: Supplementary file 1 [file ganc-08-484-s001.pdf]

Genetically transforming human osteoblasts to sarcoma: development of an osteosarcoma model – Yang et al

Supplementary Figures

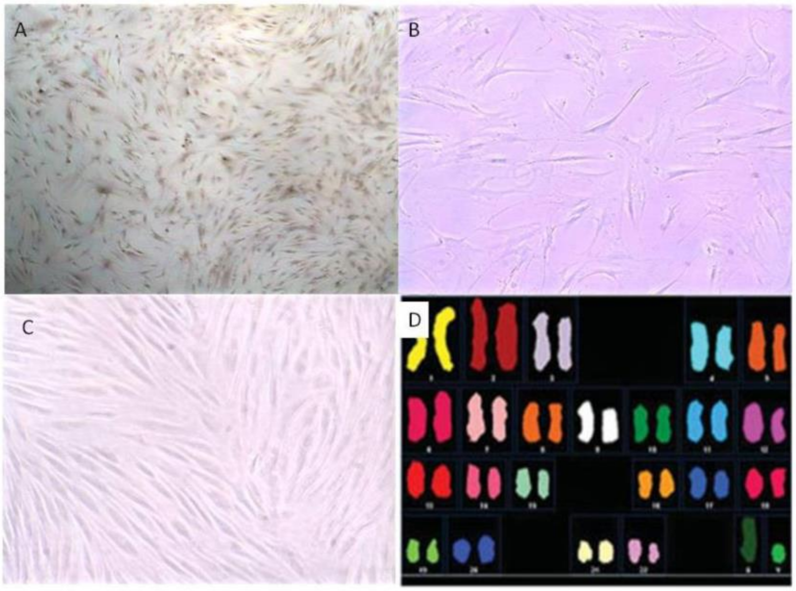

Figure S1: Morphological changes in aggregates of human mesenchymal stem cells.

Human mesenchymal stem cells (hMSCs) cultured in medium supplemented with 10% FBS (20×magnification): (A) 2 days after initial cell seeding; (B) after the first cell passage; (C) after the seventh cell passage; and (D) a normal karyotype by spectral karyotyping for hMSCs eight passages after derivation.

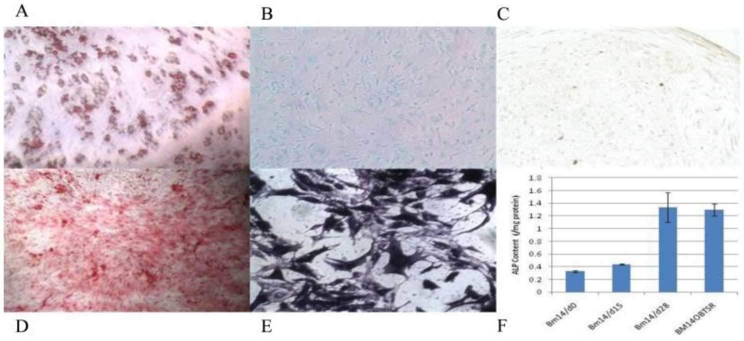

Figure S2: Images of functional differentiation of human mesenchymal stem cells. hMSCs cultured in (A) adipogenic medium with Oil Red O staining on Day 14 demonstrating red lipid droplets in cytoplasm (20×); (B) chondrogenic medium with safranin O staining of the extracellular matrix (ECM) and cells with large nuclei in lacunae on Day 21 (40×); (C) immunohistochemical staining showing patchy brown staining of collagen type II; (D) alizarin red staining of mineralized ECM on Day 21 (20×); (E) Alkaline phosphatase (ALP) staining in hMSCs in osteogenic medium on Day 14; (F) Progression of ALP activity of hMSCs in osteogenic medium for a period of 28 days. ($P < 0.01$) ($n = 3$).

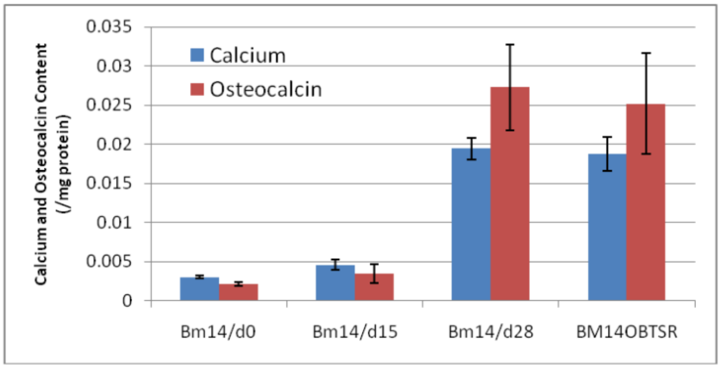

Figure S3: Osteocalcin and calcium expression of hMSCs. hMSCs were cultured in osteogenic medium for a period of 28 days and osteocalcin and calcium expression were measured. There is a significant increase in osteocalcin expression and calcium deposition from Day 0 and 14 to Day 28 ($P < 0.05$) ($n = 3$, mean ± SD). Genetic modification demonstrates minimal effect on osteoblast phenotype.

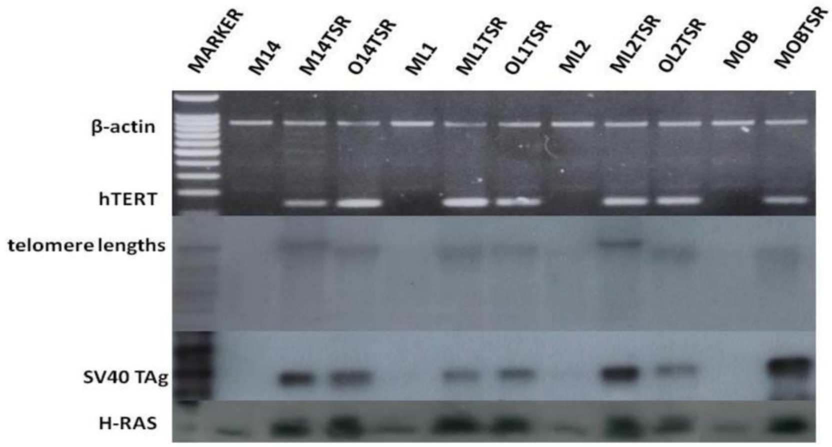

Figure S4: Expression of hTERT, SV40 TAg and H-Ras in the target cell. hTERT expression was confirmed in hMSC-TSR, MOB-TSR, and pOB-TSR by RT-PCR with β-actin serving as a control. Telomere lengths were analyzed by hybridization of genomic DNA with a telomerase-specific oligonucleotide probe. SV40 TAg and H-Ras protein expression was confirmed by western blots.

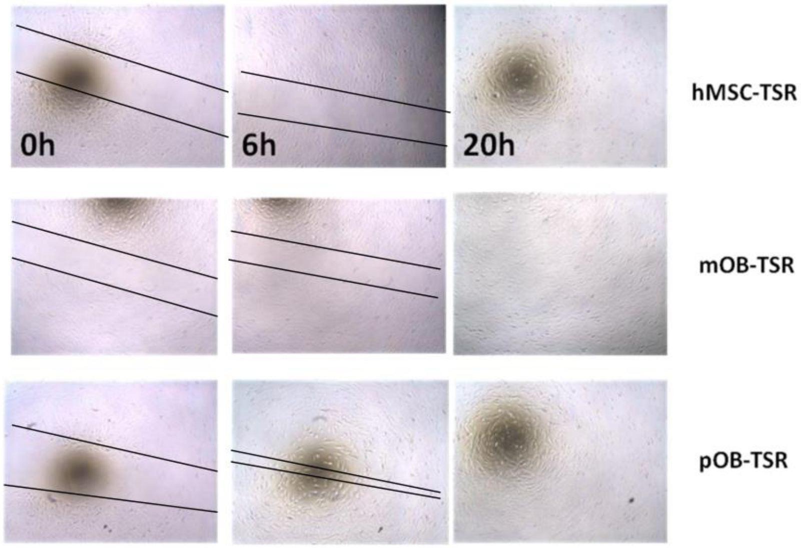

Figure S5: Wound healing assay with hMSC-TSR, mOB-TSR, and pOB-TSR cells. Wound healing assays were performed using hMSC-TSR cells, mOB-TSR cells, and pOB-TSR cells. Microscopic observations were recorded 0, 6, and 20 hours after scratching the cell surface. No significant differences were observed when comparing random and haptotaxis migration capacity of MSC-TSR, pOB-TSR and mOB-TSR.

Supplementary Tables

Table S1: Gene Expression Correlation between MicroArray and Real-time RT-PCR

		Real Time	Microarray	Correlation
OB	LPL	1	-1.11	
	FABP4	5.36	1.03	
	PPARG	1.54	1.62	
	IBSP	0.65	-1.19	
	BGLAP	0.12	1	
	SP7	0.83	0.89	
	RUNX2	0.12	1.11	0.20
MSC-TSR	LPL	4.41	3.53	
	FABP4	2.5	-1.07	
	PPARG	1.74	-2.48	
	IBSP	0.03	-3.31	
	BGLAP	0.04	-1.04	
	SP7	3.49	-1.09	
	RUNX2	3.08	-1.1	0.71
OB-TSR	LPL	21.3	-1.01	
	FABP4	14.09	1.13	
	PPARG	2.42	-2.59	
	IBSP	0.12	-3.22	
	BGLAP	0.53	-1.04	
	SP7	4.74	-1.18	
	RUNX2	0.95	-1.48	0.58

The expression levels of a panel of genes involved in osteoblastic, chondrogenic and adipogenic differentiation were measured using real-time RT-PCR. The correlation coefficients of the fold changes between real-time RT-PCR and MicroArray in these specimens as compared to the MSCs were shown in the right column.

Table S2: Phenotypic and genetic characterization of the transformed osteosarcoma cell lines.

Cell Line Abbr.	Cell type description	Phenotypic Characterization	Genetically transforming Derivatives
M14	BM-MSC (CHAM)	FACS analysis	M14-TSR
ML1	BM-MSC (LONZA)	FACS analysis	ML1-TSR
ML2	BM-MSC (LONZA)	FACS analysis	ML2-TSR
O14	PreOB Diff from M14	ALP+, OC+, Alizarin Red+	O14-TSR
OL1	PreOB Diff from ML1	ALP+, OC+, Alizarin Red+	OL1-TSR
OL2	PreOB Diff from ML2	ALP+, OC+, Alizarin Red+	OL2-TSR
mOB	Mature OB	ALP+, OC+, Alizarin Red+	mOB-TSR

Abbreviations: BM-MSC=bone-marrow-derived mesenchymal stem cells; OB=osteoblasts; Diff=differentiation; FASC=Fluorescence Activated Cell Sorting; ALP=alkaline phosphatase; OC=osteocalcin; TSR= *hTERT*, *SV40 Tag*, and H-*Ras*; CHAM=the Children's Hospital at Montefiore.

Table S3: Gene Expression Profile by Ingenuity Pathway Analysis.

Transformed vs. Control	Canonical pathway	Molecules	p-Value
	Role of BRCA1 in DNA Damage Response	46	2.01E -13
	Hereditary Breast Cancer Signaling	60	5.37E -07
	Mismatch Repair in Eukaryotes	14	5.19E -06
	Cell Cycle Control of Chromosomal Replication	20	6.07E -06
	ATM Signaling	35	8.07E -06
	Cleavage and Polyadenylation of Pre-mRNA	11	2.41E -05
	Estrogen-mediated S-phase Entry	17	7.96E -05
	Cell Cycle: G1/S Checkpoint Regulation	34	1.55E -04
	Purine Nucleotides De Novo Biosynthesis II	9	8.2E -04
	DNA damage-induced 14-3-3σ Signaling	13	9.69E -04
**Transformed vs. Xenograft**	PI3K/AKT Signaling	41	1.81E -08
	Axonal Guidance Signaling	95	1.13E -06
	Molecular Mechanisms of Cancer	75	1.5E -05
	p53 Signaling	29	2.86E -05
	NRF2-mediated Oxidative Stress Response	44	6.23E -05
	Inhibition of Angiogenesis by TSP1	14	6.86E -05
	Wnt/β-catenin Signaling	42	8.06E -05
	Glucocorticoid Receptor Signaling	58	9.02E -05
	IL-17A Signaling in Fibroblasts	14	1E -04
	Hepatic Fibrosis / Hepatic Stellate Cell Activation	35	1.82E -04
